# Synthesis and Characterization of Hydrophobically Modified Xylans

**DOI:** 10.3390/polym13020291

**Published:** 2021-01-18

**Authors:** Huai N. Cheng, Atanu Biswas, Sanghoon Kim, Carlucio R. Alves, Roselayne F. Furtado

**Affiliations:** 1 Southern Regional Research Center, USDA Agricultural Research Service, 1100 Robert E. Lee Blvd., New Orleans, LA 70124, USA; 2 National Center for Agricultural Utilization Research, USDA Agricultural Research Services, 1815 N. University Street, Peoria, IL 61604, USA; sanghoon.kim@usda.gov; 3 Chemistry Department, State University of Ceará, Silas Munguba Av. 1.700, Fortaleza, CE 60740-020, Brazil; alvescr@yahoo.com; 4 Embrapa Agroindústria Tropical, Rua Dra. Sara Mesquita 2270, Fortaleza, CE 60511-110, Brazil; roselayne.furtado@embrapa.br

**Keywords:** alkyl ketene dimer, alkenyl succinic anhydride, hydrophobic modification, octenyl succinic anhydride, tetrapropenyl succinic anhydride, xylan

## Abstract

Xylan is a major type of hemicellulose that has attracted a lot of research and development activities. It is often derivatized in order to improve its properties. In the literature, hydrophobic modification of polymers is often used to produce surfactant-like materials and associative thickeners. In this work, we have derivatized xylan with alkyl ketene dimer (AKD) and two types of alkenyl succinic anhydrides (ASAs). The xylan-AKD derivatives have been made at 90 °C, using dimethyl sulfoxide as solvent and 4-dimethylaminopyridine as promoter. Samples with degrees of substitution (DS) up to 0.006 have been produced. The xylan-ASA derivatives have been synthesized at 120 °C in dimethyl sulfoxide with DS up to 0.105–0.135. The structures of these products have been confirmed with NMR and FT-IR. These xylan derivatives increase the structural diversity of xylan and provide additional options for people seeking to use hydrophobically modified polysaccharides in their applications.

## 1. Introduction

Hemicellulose is among the most abundant polysaccharides in nature and is found in almost all plant cell walls [1,2,3]. Xylan is a major type of hemicellulose, consisting of β-1,4-linked xylose residues with side branches of α-arabinofuranose and α-glucuronic acids. It is the main hemicellulosic component of hardwoods and accounts for approximately 30% of the woody cell wall. It is also found in many other plants, such as cotton, sugar cane, wheat straw, sorghum, and corn. Because xylan is abundantly available, there has been a lot of effort [1,2,3,4,5,6,7] in the past several years to exploit it as a sustainable bio-resource for new product development. Because it is under-utilized, interest has been shown to modify it with different functional groups to improve its properties; several excellent reviews [4,5,6,7] are available.

Hydrophobically modified polymers have attracted a fair amount of attention, either for their use in soluble solutions or in insoluble hydrogels [8,9,10,11]. In solution, they are valued for their surfactant properties and rheology control. The insoluble hydrogels can be utilized for controlled drug release applications. Structurally, these are water-soluble polymers with hydrophobic side chains or end groups. The hydrophobic groups can associate with one another in an aqueous medium, exhibiting a shear-thinning rheological behavior. Common water-soluble polymers include poly(ethylene glycol), acrylic polymers, or polysaccharides. Hydrophobically modified polysaccharides are of particular interest because they are biobased, and many of them have been reported [12,13,14,15,16]. For example, they have been used for rheology modification [12,13,16], enzyme immobilization [17] and drug encapsulation [18,19].

Being hydrophilic, xylan has often been modified with hydrophobic moieties [6,7,15]. particularly for packaging and coating applications. For example, esterification is among the most frequently used method, and there has been many publications on acetylated xylan e.g., [20,21,22,23,24,25]. Moreover, xylan esters with longer alkyl chains [26,27,28], and benzylated xylan [29] have also been reported. Another approach is to react xylan with propylene oxide and then to acetylate [30], butylate or allylate [31] the hydroxypropyl xylan; these modifications were reported to impart hydrophobicity to xylan. A different approach is to fluorinate xylan, e.g., through the addition of trifluoroacetic functionality [32]. Another reported approach (described below) is the reactions of xylan with an anhydride. In view of the continuing interest in this field, new hydrophobically modified xylans may be of interest from both fundamental and practical points of view.

Alkyl ketene dimers (AKDs) are fairly well-known sizing agents for paper applications [33,34]. An AKD can be made from a fatty acid chloride in an inert solvent with a tertiary amine under anhydrous conditions. A hydroxyl group can react with it to form a β-keto ester. In paper sizing, at least some of the AKD can react with the cellulose chains on paper to form covalent bonds [35,36]. There has been recent interest in reacting AKD with different types of polysaccharides, e.g., microcrystalline cellulose [37], microfibrillated cellulose [38,39], cellulose nanofibers [40,41], bacterial cellulose [42], carboxymethyl cellulose [43], hydroxyethyl cellulose [44], starch [45,46], and cashew gum [47]. Thus far, the reaction of xylan with a ketene dimer has not been reported.

Alkenyl succinic anhydrides (ASAs) are also being used as sizing agents for paper. Thus, an ASA reacts with hydroxyl groups of the cellulose to form an ester, anchoring the hydrophobic group to the paper surface. It is prepared by the ene reaction of unsaturated hydrocarbons with maleic anhydride [48,49]. Two often-used ASAs are octenyl succinic anhydride (OSA) and dodecyl succinic anhydride (DDSA). Studies involving starch-OSA adducts have been reviewed recently by Altuna et al. [50] and Sweedman [51]. A review of modifications with DDSA has been given by Shah [52]. Several papers on the reaction of xylan and ASA have been reported [53,54,55,56], including the synthesis in an ionic liquid [53], water [56], dimethylsulfoxide (DMSO)-triethylamine [54], and DMSO with 2M HCl addition for pH adjustment [55]. In addition to starch and xylan, ASAs have been used to modify other polysaccharides, such as cashew gum [57], inulin [58], and tamarind seed xyloglucan [59].

A key consideration in the applications of hydrophobic polymers is the size of the hydrophobe relative to the size and nature of the polymer. It is useful, therefore, to study long-chain alkyl substituted xylans with different lengths in the alkyl units. In this work, we have reacted xylan with two more hydrophobic reagents (AKD and TPSA), thereby generating two new hydrophobically modified xylans. These new additions provide more options and diversity for future use. The inclusion of OSA (with 8 carbons) in this work also permits comparisons to be made with TPSA (12 carbons) and AKD (about 32 carbons). An advantage of the use of AKD and TPSA is their commercial availability; thus, the new hydrophobically modified xylan can be produced commercially if needed.

## 2. Materials and Methods

### 2.1. Materials

Xylan was purchased from three sources: Lenzing AG (Lenzing, Austria), Sigma-Aldrich (St. Louis, MO, USA), and Megazyme Inc. (Chicago, IL, USA). Our initial work was done with Sigma and Megazyme products; however, we reported herein only the data involving the Lenzing material. DMSO and isopropanol were purchased from Fisher Scientific (Pittsburgh, PA, USA). Absolute ethanol was acquired from Decon Laboratories, Inc. (King of Prussia, PA, USA). D_2_O and d_6_-DMSO were obtained from Cambridge Isotope Laboratories (Andover, MA, USA). AKD (Aquapel^TM^ 364) was a gift, courtesy of Solenis, LLC (Wilmington, DE, USA). Both OSA and TPSA samples were bought from Milliken Chemical Company (Spartanburg, SC, USA).

### 2.2. Synthesis of Xylan Derivatives

For the synthesis of xylan-AKD adducts, the xylan was dried overnight in a vacuum oven at 70–80 °C before use. Typically, 1 g of xylan, 0.02–0.21 g of AKD, and 0.01–0.10 g of 4-dimethylaminopyridine (DMAP) were dissolved in 4 mL DMSO and heated at 90 °C for 3 h with stirring in a Reacti-Therm^TM^ reactor (Fisher Scientific, Pittsburgh, PA, USA). At the completion of the reaction, the mixture was then cooled down, and isopropanol added dropwise with stirring to prevent any large clumps from forming. The precipitate was filtered and washed with methylene chloride for 30 min to remove excess AKD. The final product was vacuum filtered and dried under vacuum at 90 °C overnight.

In order to synthesize xylan-ASA derivatives, we dissolved 1 g xylan (previously dried overnight at 70–80 °C) and 0.02–0.20 g ASA in 1.5–4.0 mL DMSO. The reaction mixture was placed in a Reacti-Therm^TM^ set-up, heated with stirring at 120 °C for 3–5 h, and then cooled down. Isopropanol was added slowly with stirring to precipitate the product while minimizing the formation of large clumps. The product was washed successively with isopropanol and methylene chloride, filtered, and dried overnight in a vacuum oven at 90 °C.

### 2.3. Polymer Characterization

For the acquisition of NMR spectra, a Bruker Avance 500 spectrometer (Carlstadt, Germany) was employed with a 5 mm broadband observe probe. About 50–200 mg of each modified xylan sample were dissolved in 1 g d_6_-DMSO. The ^1^H spectrum was obtained at 27 °C under standard instrumental conditions and processed with Bruker Topspin 1.3 pl 8 software.

For Fourier transform infrared (FT-IR) measurements, we used a Nicolet iS10 spectrometer (Thermo Scientific Inc., Waltham, MA, USA) with a DTGS detector and KBr beam splitter. The sample was placed on an ATR diamond crystal and attached to a Smart Orbit single bounce ATR accessory. The instrumental conditions included: 600–4000 cm^−1^ spectral region, 32 scans per spectrum, 4 cm^−1^ resolution, and room temperature. Data processing was accomplished with the Omnics software (version 9.2.98).

Thermogravimetric analyses (TGA) and differential TGA (DTG) were done under N_2_ on a Model Q500 analyzer (TA Instruments, New Castle, DE, USA). Each sample (~3 mg) was heated from 25 °C to 600 °C at a rate of 10 °C /min. Data were processed with TA Instruments Universal Analysis 2000 software.

For size exclusion chromatography (SEC), the instrument set-up included a Shimadzu Prominence LC system (Kyoto, Japan) together with refractive index and UV diode-array detectors, and a Phenogel 5 μm Linear SEC column (Phenomenex, Torrance, CA, USA), operating at 60 °C. The mobile phase was DMSO. Each sample was dissolved in the mobile phase at 0.5% and filtered through a 0.45 μm syringe filter. For each analysis, 50 μL of the sample solution was injected into the SEC unit. The flow rate was 0.5 mL/min. Molecular weight calibration was made using three dextran standards (5 kDa, 25 kDa, and 670 kDa). As the employed column was linear in the MW range of 100 to 10M Da, the elution time could be converted to MW after the construction of calibration curve.

## 3. Results and Discussion

### 3.1. Reaction of Xylan with AKD

**Scheme 1:**
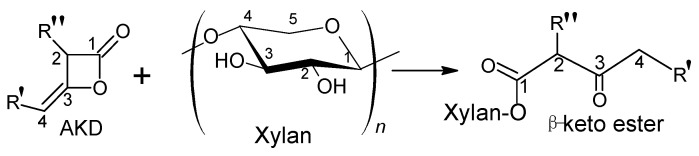
Reaction of AKD with xylan, where R′ and R″ are C_14_H_29_. The reaction of xylan with AKD can occur at OH positions 2 and/or 3 of xylan.

The reaction of AKD is shown in Scheme 1. In view of our previous experience with AKD reactions [47], we chose DMSO as the solvent for this reaction and optimized the lab procedures for higher yields.

**Figure 1:**
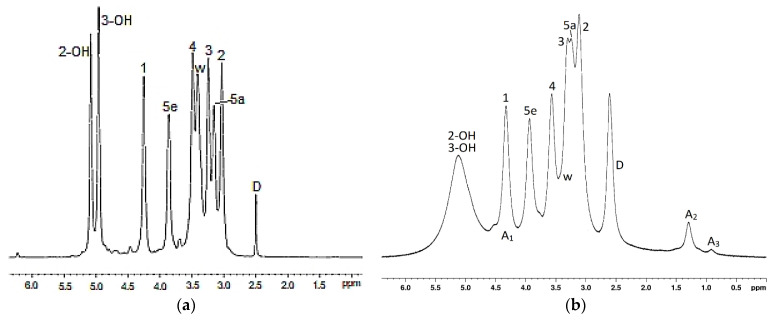
^1^H NMR spectra of (**a**) unreacted xylan and (**b**) xylan-AKD derivative A4 in d_6_-DMSO; D = DMSO, and w = water peak. The xylan peaks are numbered according to Scheme 1.

The ^1^H NMR spectrum of a xylan-AKD sample is shown in Figure 1a. The xylan gave multiple peaks, and the assignments were reported earlier [60,61,62]. These assignments are noted in Figure 1a. The xylan 2-OH and 3-OH peaks occurred at 5.08 ppm and 4.95 ppm, respectfully. Further upfield were H1 (4.35 ppm), H5e (3.86 ppm), H4 (3.48 ppm), water peak (3.3–3.9 ppm), H3 (3.22 ppm), H5a (3.16 ppm), and H2 (3.01 ppm). The ^1^H NMR spectrum of a xylan-AKD adduct is shown in Figure 1b; the peaks showed broader linewidths than those of xylan because the AKD-xylan solution exhibited increased viscosity. In the spectrum for the xylan-AKD adduct, two distinct groups of peaks showed up at ca. 1.1–1.65 ppm, corresponding to the long-chain methylene groups, and at 0.8 ppm, corresponding to the methyl groups; these assignments are consistent with the literature values [45,47].

Because of the presence of the water peak, which moved around at ca. 3.3–3.5 ppm, we needed to use the peak areas for 2-OH, 3-OH, H1 and H5e for the estimation of DS. Let the average peak area for these four peaks be A_1_, corresponding to the molar amount of xylan present. For the xylan-AKD derivative, since there were about 52 methylene protons that resonated at ca. 1.1–1.65 ppm (peak area A_2_), the molar amount of AKD residue corresponded to this peak area divided by 52; thus one way to calculate the DS was A_2_/(52 × A_1_). A second way to calculate DS was to use the methyl peak at 0.8 ppm. Since each AKD molecule contained two methyl groups and each methyl had three hydrogens, the methyl area (A_3_) needed to be divided by six. Thus, the DS could be estimated via the following expression: A_3_/(6 × A_1_). The average of these two calculations gave the reported DS value.

Several xylan-AKD derivatives were prepared as shown in Table 1. As we increased the amount of AKD in the reaction, the DS increased, as expected. When the samples were dissolved in DMSO, all the sample solutions became turbid and more viscous. As the solutions were heated to 60 °C, they all cleared up. When the solutions were cooled back down, samples A1 and A2 stayed clear, but samples A3 and A4 remained somewhat turbid. These observations were due to the aggregation of the hydrophobic groups in the xylan-AKD adduct and were more apparent at higher DS values of AKD, causing increasing aggregation of the hydrophobic groups. The same observation of solution opacity was also reported for AKD derivatives of starch [45] and cashew gum [47].

**Figure 2:**
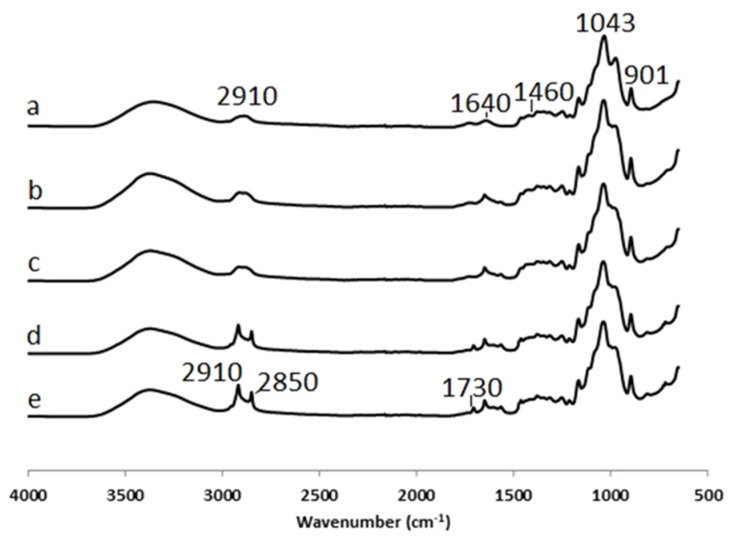
FT-IR spectra of xylan and xylan-AKD adducts: (**a**) unreacted xylan, (**b**) sample A1, (**c**) sample A2, (**d**) sample A3, and (**e**) sample A4.

The FT-IR spectra of xylan and xylan-AKD adducts are shown in Figure 2. The xylan spectrum (Figure 2a) was similar to what were published earlier [63,64,65,66,67,68,69,70,71]. The broad feature at around 3400 cm^−1^ was due to O–H stretching, and the 2910 cm^−1^ peak to C–H vibrations. The weak peak at ca. 1640 cm^−1^ was caused by residual water in the sample [67,70]. The 1460 cm^−1^ peak came from CH and OH bending in xylan [67,69]. The large peak at 1043 cm^−1^ was attributed to C–O stretching in C–O–C linkages [65,67]. The peak at 901 cm^−1^ corresponded to C1–H bending and ring frequency modes in β-glycosidic linkages between the xylose units [66,69,70]. In the FT-IR spectra of xylan-AKD adducts (Figure 2b–e), the C-H vibration peaks at 2920 and 2850 cm^−1^ increased in intensities due to the large number of CH bonds in the xylan-AKD structure. Moreover, the peaks at ca. 1720–1740 cm^−1^ showed up, corresponding to the C=O stretch of ester and the ketone.

**Figure 3:**
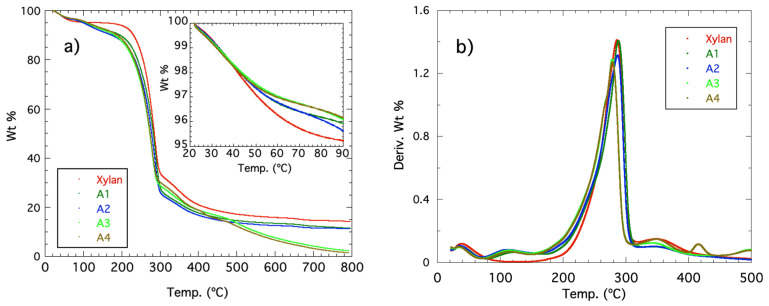
(**a**) TGA and (**b**) DTG curves in nitrogen for xylan and xylan-AKD derivatives.

The TGA and DTG curves obtained in nitrogen are given in Figure 3. All the samples showed loss of water at about 25–100 °C. The weight of water loss in xylan-AKD adducts was slightly lower than that of xylan (Figure 3a, inset), suggesting that hydrophobic modification slightly reduced the amount of water adsorbed to xylan [57]. Moreover, for xylan-AKD adducts, somewhat greater weight loss was found at about 120–200 °C; this difference seemed to occur also with the starch-AKD derivative relative to unreacted starch [46]. Most of the degradation of xylan backbone and ester cleavage took place at about 200–295 °C. For xylan-AKD adducts with increasing AKD, the maximum on the DTG curve at ca. 280 °C shifted to slightly lower temperatures, indicating slightly decreased thermal stability of the xylan-AKD derivatives, relative to the unreacted xylan.

At higher temperature (>300 °C), the TGA curves for samples A1 and A2 appeared similar, with about 4–7% less residual weight relative to xylan. However, samples A3 and A4 showed noticeably lower char yields above 500 °C, perhaps because the AKD substitution caused disruption of the unsubstituted xylan structure, thereby reducing the amount of char formed.

### 3.2. Reaction of Xylan with OSA

**Scheme 2:**
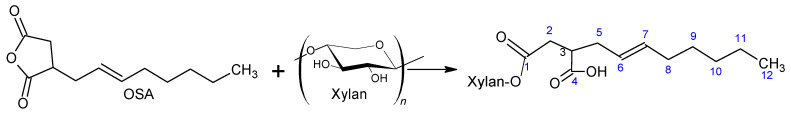
Schematic reaction of xylan with OSA. Substitution of OSA can occur at OH positions 2 and/or 3 of xylan. On the succinic moiety, the xylan can be attached to carboxyl in position 1 (as shown) or in position 4.

The reaction scheme for xylan and OSA is shown in Scheme 2. We continued to use DMSO as a solvent for this reaction, and products that formed clear solutions in DMSO were obtained. We found workable reaction conditions that entailed 120 °C reaction temperature and 3 h reaction time (Table 2).

**Figure 4:**
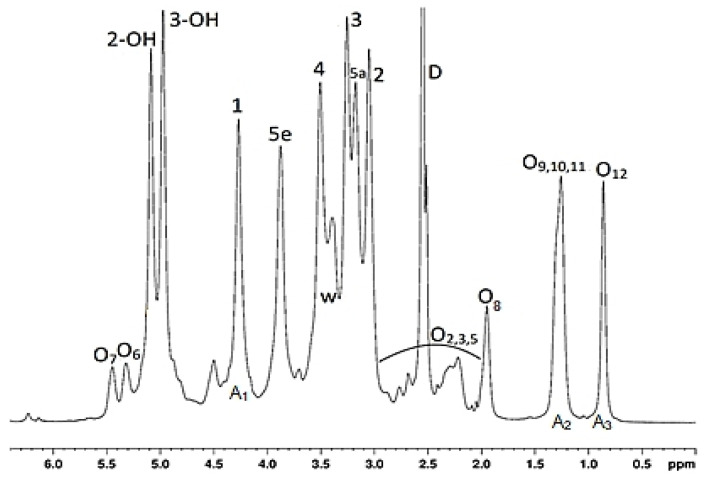
^1^H NMR spectrum of xylan-OSA derivative B5 in d_6_-DMSO; D = DMSO, w = water peak. The peak numbers for OSA (O) follow the numbering scheme in Scheme 2**.**

The ^1^H NMR spectrum for xylan-OSA is given in Figure 4. Through the literature [51,53,55,72] and our previous work [57], we can make the assignments, as noted in Figure 4. The molar amount of xylan can be estimated from the peak area at either 4.3 ppm (H1, 1 proton) or 3.95 ppm (H5e, 1 proton); let the average area of these two peaks be A_1_. The peak area at 2.0 ppm (O8, 2 protons), 1.3 ppm (O9 + O10 + O11, 6 protons, area = A_2_), or 0.85 ppm (O12, 3 protons, area = A_3_) provides the molar amount of OSA present. The DS can be estimated by A_2_/(6 × A_1_) or A_3_/(3 × A_1_). These estimated DS values are shown in the last column of Table 2. From the data, about 70–100% of OSA have reacted with xylan.

**Figure 5:**
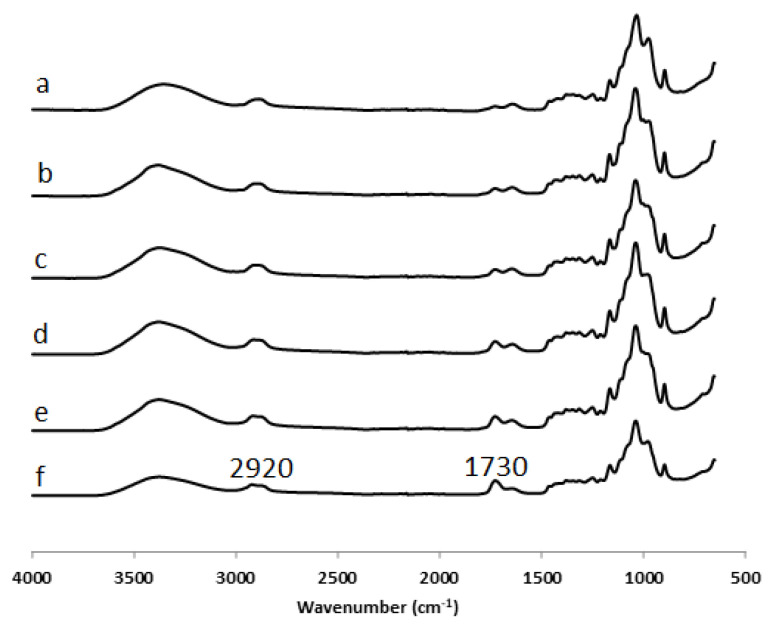
FT-IR spectra of xylan and xylan-OSA adducts: (**a**) unreacted xylan, (**b**) sample B1, (**c**) sample B2, (**d**) sample B3, (**e**) sample B4, and (**f**) sample B5.

The FT-IR spectra of xylan and xylan-OSA derivatives are shown in Figure 5. When compared to the xylan spectrum, the peak at 1730 cm^−1^ stood out; it corresponded to the ester C=O stretch [53,54,55,56]. With increasing OSA level, this peak became larger. Moreover, the C–H vibration peak at 2920 cm^−1^ became slightly larger with increasing OSA levels.

**Figure 6:**
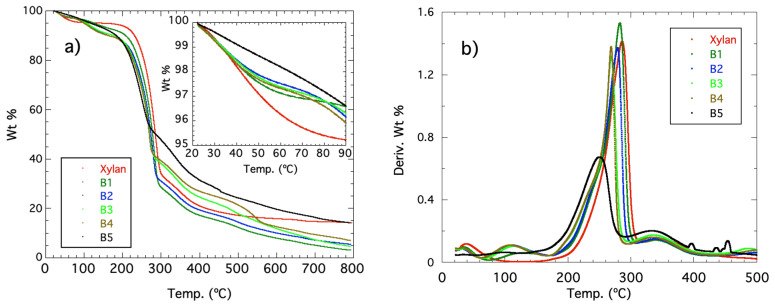
(**a**) TGA and (**b**) DTG curves in nitrogen for xylan and xylan-OSA samples.

The TGA data are shown in Figure 6a. As in the case of xylan-AKD derivatives, the weight loss of water in xylan-OSA adducts at 25–100 °C was somewhat less than that of xylan (Figure 6a, inset). This suggested that less water was adsorbed by OSA-modified xylans; similar results were found earlier for OSA-modified cashew gum [57]. Furthermore, a greater weight loss for the xylan-OSA derivatives was found, relative to xylan, at about 120–200 °C; this finding was similar to an earlier report on three xylan-ASA derivatives [53].

In Figure 6b, the peaks in the DTG curves shifted from 280 °C for xylan to ca. 240 °C for sample B5, indicating reduced thermal stability with OSA addition; this result confirmed past observations [53,54,55]. In samples B1–B3, lower char yields were found above 500 °C, probably because the OSA addition caused some disruption of the unsubstituted xylan structure, thereby reducing the formation of char. Nevertheless, for samples B3–B5, significantly higher residual weights were observed at ca. 295–530 °C. In order to rationalize this observation, we needed to examine the thermal degradation of xylan itself. In agreement with previous work [55], our data indicated that xylan degraded in two steps, the first step (200–295 °C) could be assigned to the cleavage of glycosidic bonds and loss of side-chain sugars [55]. The second step (ca. 295–380 °C) could be attributed to a combination of competitive dehydration, fragmentation, and disproportionation reactions [55,73], where the dehydration reactions could be catalyzed by acidic conditions [73]. Perhaps the larger amounts of succinic acid functionality in sample B3–B5 might preferentially enhance the dehydration reactions, leading to the formation of furan and other aromatic species, thereby resulting in higher residual weights at higher temperatures.

### 3.3. Reaction of Xylan with TPSA

**Scheme 3:**
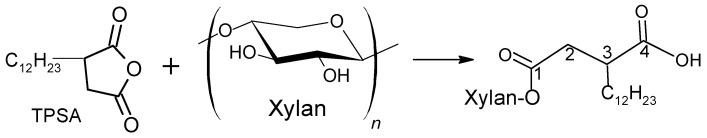
Schematic reaction of xylan and TPSA. The TPSA reaction can occur at OH positions 2 and/or 3 of xylan. On the succinic part, the xylan can be at carboxyl position 1 (as shown) or position 4.

The reaction of xylan with TPSA is shown schematically in Scheme 3. As noted earlier [57], TPSA has a complex structure because it is derived from propylene tetramer, which is a mixture of different isomers. For convenience, the propylene tetramer structure is abbreviated as C_12_H_23_ in Scheme 3.

We again adopted DMSO as the solvent but needed to use less DMSO (1.5 mL) together with 5 h reaction time and 120 °C reaction temperature (Table 3). The products gave clear solutions in DMSO.

**Figure 7:**
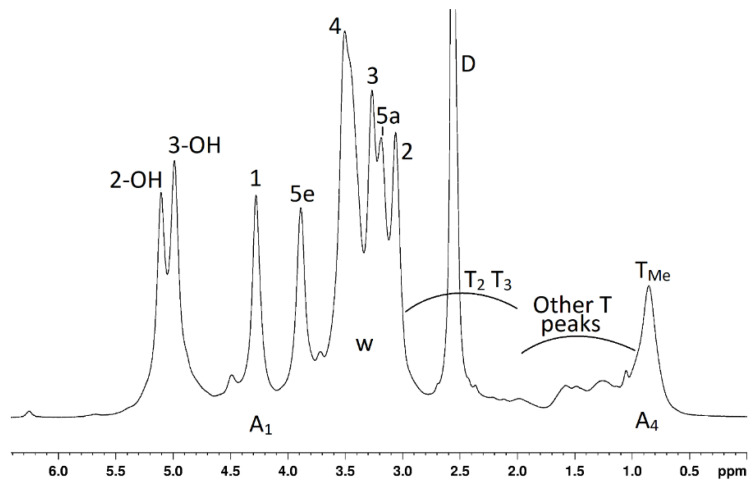
^1^H NMR spectra of xylan-TPSA sample C5 in d_6_-DMSO. D = DMSO, w = water peak. The peak numbers for TPSA (T) follow the numbering scheme in Scheme 3.

The ^1^H NMR spectrum of a xylan–TPSA product is shown in Figure 7. The peaks for unreacted xylan were the same as in Figure 1 and Figure 4. In view of the complexity of the TPSA structure, only general peak assignments for TPSA residue are noted in Figure 7. For the estimation of DS, we used the methyl peak at 0.8 ppm (peak area A_4_). Since there were 12 protons in the four (non-allylic) methyl groups, the area per mole for the TPSA residue was A_4_/12. We used xylan H1 and H5e peaks (with average area of A_1_) for the molar amount of xylan. The DS could be estimated via the expression: A_4_/(12 × A_1_). The estimated DS values are shown in the last column of Table 1. About 70–80% of TPSA have reacted with xylan.

**Figure 8:**
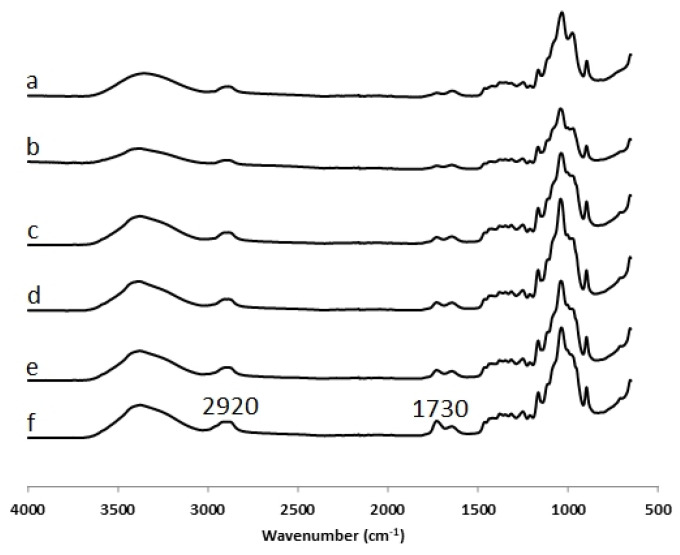
FT-IR spectra of xylan and xylan-TPSA adducts. (**a**) unreacted xylan, (**b**) sample C1, (**c**) sample C2, (**d**) sample C3, (**e**) sample C4, and (**f**) sample C5.

In Figure 8 are given the FT-IR spectra of xylan and xylan–TPSA adducts (samples C1–C5). The spectrum for unreacted xylan (Figure 8a) was the same as before. The spectra of xylan-TPSA derivatives (Figure 8b–f) showed progressively larger peaks at 1730 cm^−1^ (ester) and 2920 cm^−1^ (C–H vibration peak). These observations were consistent with the increasing formation of the xylan-TPSA derivative. Thus, the FT-IR data corroborated the NMR observations and confirmed that the reaction had occurred.

**Figure 9:**
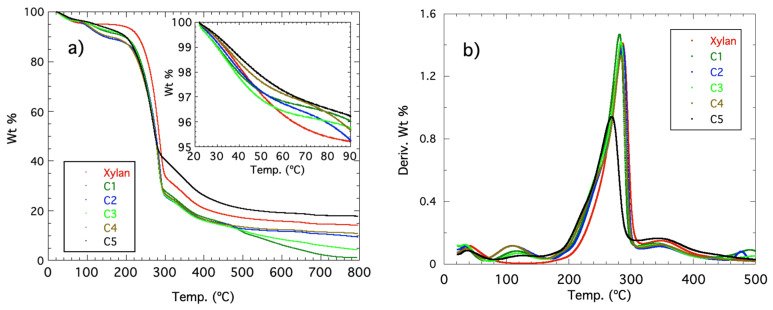
(**a**) TGA and (**b**) DTG curves in nitrogen for xylan and xylan-TPSA samples.

The TGA and DTG data for xylan and xylan-TPSA adducts are shown in Figure 9. Here, the trend for the weight loss of water at 25–100 °C for xylan-TPSA relative to xylan (Figure 9a, inset) was not as clear-cut as in Figure 6a. As in the case of xylan-OSA derivatives, xylan-TPSA samples lost more weight than xylan at about 120–200 °C; this was also observed earlier for three other xylan-ASA derivatives [53]. At the xylan’s main degradation region of 200–295 °C, the maximum in the DTG curves decreased from 280 °C for xylan to ca. 260 °C for sample C5. Thus, TPSA substitution decreased the thermal stability of xylan. In samples C1–C4, there were notably lower residual weights above 295 °C. However, for samples C5, a higher residual weight was found at >295 °C, perhaps due to preferential dehydration of the depolymerized xylan fragments that was catalyzed by the larger amount of carboxylic functionality in the TPSA moiety in sample C5. This observation was similar to those of the high DS xylan-OSA adducts shown in Figure 6a.

### 3.4. SEC Analysis

**Figure 10:**
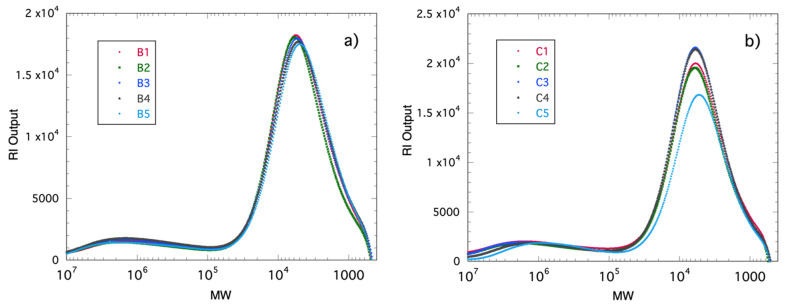
SEC data for (**a**) xylan-OSA derivatives, (**b**) xylan-TPSA derivatives.

We attempted to carry out the SEC experiment on the xylan derivatives. The SEC chromatograms for xylan-OSA and xylan-TPSA derivatives are shown in Figure 10.

From the SEC data, the xylan samples appeared to contain two components with different molecular weights. In the literature, some xylan samples were indeed reported to contain more than one molecular weight component [53,74,75,76]. It is noteworthy that there was no significant changes in molecular weight due to the derivatization reactions. Similar findings were made for the OSA and TPSA derivatives of cashew gum [57].

An attempt was made to obtain the SEC data for xylan-AKD derivatives. Because AKD contained two long-chain alkyl units, it was especially hydrophobic, and the propensity for aggregation was strong. As a result, we were not able to get usable SEC data from the xylan-AKD derivatives. This finding was consistent with our observation that xylan-AKD derivatives tended to form turbid solutions in DMSO due to aggregation. In contrast, the xylan-OSA and xylan-TPSA derivatives gave mostly clear solutions in DMSO.

## 4. Conclusions

Since xylan is available in nature and is currently under-utilized, it has been derivatized in this work with three hydrophobic reagents (AKD, OSA, and TPSA), which can impart an interfacial or surfactant-like property to xylan. All three reactions have been shown to proceed in DMSO at high temperatures. Two of these reagents have not previously been used with xylan, and the resulting products are new. Several samples with varying DS values have been made, and the chemical structures have been verified with NMR and FT-IR. From the turbidity observations, the strength of hydrophobicity follows this trend: AKD > TPSA > OSA. In other words, the more alkyl carbons the xylan derivative contains, the more hydrophobic it becomes.

In the development of formulations involving hydrophobic polymers, it is known that the chain length has a significant impact on the desired end-use functions. For example, hydrophobically modified hydroxyethyl celluloses with different hydrophobe lengths and types exhibit different adsorption to acrylic latex, resulting in different paint behavior [77]. Likewise, hydrophobically modified alkali-soluble emulsion polymers show different degrees of association depending on the hydrophobe size [78]. Thus, it is helpful to have different types of hydrophobically modified polymer available, and the best material can then be selected for a given application. The two new hydrophobically modified xylans disclosed herein may be useful additions to the family of hydrophobically modified polymers in this context.

## Figures and Tables

**Table 1:** Degree of substitution of xylan-AKD products obtained with 1 g xylan, 4 mL DMSO at 90 °C and 3 h reaction temperature.

Sample #	Xylan (g)	AKD (g)	AKD/xyl Mol Ratio	DMAP (g)	Weight Yield (g)	DS
A1	1	0.02	0.0026	0.01	0.998	0.0013
A2	1	0.05	0.0066	0.02	1.037	0.0019
A3	1	0.156	0.0206	0.075	1.044	0.0036
A4	1	0.208	0.0274	0.1	1.078	0.0059

**Table 2:** Reactions of 1 g xylan with OSA in 4 mL DMSO at 120 °C with a 3 h reaction time.

Sample	Weight of OSA, g	Product Weight, g	Weight Yield %	Obsd DS
B1	0.02	0.9977	98	0.016
B2	0.04	1.0391	99	0.036
B3	0.084	1.0113	93	0.052
B4	0.104	0.797	72 *	0.059
B5	0.2	1.091	91	0.135

* Part of sample lost during transfer.

**Table 3:** Reactions of 1 g xylan with TPSA in 1.5 mL DMSO at 120 °C with a 5 h reaction time.

Sample	Weight of TPSA, g	Product Weight, g	Weight Yield %	Obsd DS
C1	0.02	0.9548	94	0.010
C2	0.04	1.0057	97	0.018
C3	0.084	1.0064	93	0.039
C4	0.104	0.9888	90	0.052
C5	0.2	0.9833	82	0.105

## Data Availability

No further data were analyzed in this study. Data sharing is not applicable to this article.
